# Polypharmacy among people diagnosed with colorectal cancer in Australia: a population-based cohort study

**DOI:** 10.1093/oncolo/oyaf380

**Published:** 2025-12-09

**Authors:** Benjamin Daniels, Monica Tang, Maria Aslam, Sallie-Anne Pearson

**Affiliations:** Medicines Intelligence Research Program, School of Population Health, UNSW Sydney, Sydney, NSW 2052, Australia; Medicines Intelligence Research Program, School of Population Health, UNSW Sydney, Sydney, NSW 2052, Australia; Nelune Comprehensive Cancer Centre, Prince of Wales Hospital, Sydney, NSW 2052, Australia; School of Clinical Medicine, Faculty of Medicine and Health, UNSW Sydney, Sydney, NSW 2052, Australia; Maitland Hospital, Hunter New England Local Health District, NSW 2323, Australia; Hunter Medical Research Institute, University of Newcastle, NSW 2305, Australia; School of Medicine and Public Health, College of Health, Medicine and Wellbeing, University of Newcastle, Newcastle, NSW 2308, Australia; Medicines Intelligence Research Program, School of Population Health, UNSW Sydney, Sydney, NSW 2052, Australia

**Keywords:** colorectal neoplasms, polypharmacy, health services, aged, Australia

## Abstract

**Background:**

Polypharmacy is associated with increased risks of adverse outcomes in people with cancer and medicine use evolves over the course of the disease trajectory. We estimated the prevalence of polypharmacy in people with colorectal cancer (CRC), stratified by extent of disease spread at diagnosis, during each year from 1 year prior to 5 years following diagnosis and determined factors associated with polypharmacy at diagnosis and 2- and 5-year postdiagnosis.

**Patients and Methods:**

Our retrospective cohort study used linked administrative health records from New South Wales (NSW), Australia to examine medicine use in all NSW residents ≥18 years diagnosed with CRC between 2013 and 2017. We defined polypharmacy as concomitant exposure to ≥5 medicines, excluding antineoplastics, during at least one 90-day quarter in each year from diagnosis. We used logistic regression to examine associations between polypharmacy and relevant factors.

**Results:**

Of 19 056 people diagnosed with CRC, 6797 (35%), 8482 (45%), and 3777 (20%) were diagnosed with localized, regional, and metastatic disease, respectively. Within these groups, 74%-79% experienced polypharmacy at any time during the study period; with nearly 50% experiencing polypharmacy 2 through 5 years from diagnosis. Age (≥70 years), female sex, comorbidities, colon cancer diagnosis, and socioeconomic disadvantage were associated with increased likelihood of polypharmacy at all landmarks.

**Conclusion:**

Polypharmacy is highly prevalent among Australians with CRC. For those treated with curative intent, higher polypharmacy rates are concerning, indicating excess morbidity and impaired quality of life among survivors cured of cancer but who may continue to experience poorer health than the general population.

Implications for Practice Our study highlights that rates of polypharmacy among Australians with CRC are elevated and higher than those reported in the Australian population and similar international populations. Our findings indicate that many people come to CRC diagnosis already receiving multiple medicines, reflecting a high burden of comorbidity. Our study found that medicine use changes following diagnosis and primary cancer treatment, with increased use of analgesic and psychotropic medication over time. Quality survivorship care should address the unique challenges arising post-CRC diagnosis, such as ongoing neuropathy, pain or psychological symptoms, and prioritize prevention and management of non-cancer comorbidity.

## Introduction

Colorectal cancer (CRC) is among the most commonly diagnosed cancers, worldwide.^1^ In Australia, CRC was the fourth most commonly diagnosed cancer in 2019 and was estimated to comprise 9% of all cancer diagnoses during 2023.^2^ CRC remains predominantly a disease of advanced age,^3^ however, the incidence of early-onset CRC—typically defined as diagnoses in people less than 50 years in age—has grown steadily over the past 40 years.^4^

Medicine use in people with CRC evolves over the course of their disease trajectory. Medicine use prior to a CRC diagnosis typically reflects baseline comorbidities but may also capture increasing symptom burden in the lead up to diagnosis. Medicine use postdiagnosis centers around anticancer therapies and supportive care, including management of cancer symptoms, anticancer treatment side effects and long-term sequelae. “Polypharmacy” in people with CRC, typically defined as concurrent exposure to 5 or medicines,^5^ has been associated with increased risks of potentially inappropriate medicine use, adverse events, and death.^6–10^ These findings have come from studies focused on the risks of polypharmacy in the context of active cancer treatment, often restricting cohorts to those receiving specific treatments (e.g., surgery, chemotherapy). Polypharmacy during post-treatment survivorship is not well described and little is known about medicine use amongst people diagnosed with CRC in Australia.

We aimed to describe medicine use in people with a primary CRC diagnosis. Specifically, we detailed the number and types of medicines dispensed to patients from the year preceding diagnosis, through active treatment, and into later survivorship. We determined the proportion of the cohort experiencing polypharmacy; summarized receipt of chemotherapy and other nonpharmaceutical CRC treatments; and determined the factors associated with polypharmacy at different landmark points around CRC diagnosis.

## Methods

### Setting and data

The Australian healthcare setting and datasets used in this study have been described in the Medicines Intelligence Data Platform (MedIntel DP) research protocol.^11^ Briefly, Australia maintains a publicly funded, universal healthcare system entitling citizens and permanent residents to a range of subsidized health services including hospital care and subsidized prescription medicine access through the Pharmaceutical Benefits Scheme (PBS). Cancer is a notifiable disease in Australia (excepting basal and squamous cell carcinomas), and each state maintains records of all invasive primary cancer cases.

We used data from the MedIntel DP, a linked data collection that includes administrative health records for all adult residents of New South Wales (NSW), Australia. NSW is the most populous Australian state, with approximately 6.3 million adult residents in 2020.^12^ The linked datasets include cancer notifications from the NSW Cancer Registry (NSWCR; date of diagnosis, topography, extent of disease spread at diagnosis, date of birth, sex, location of residence at time of diagnosis); PBS dispensing records (dispensed medicines, quantity dispensed, and date of dispensing for all prescription medicines); hospitalization data from the NSW Admitted Patient Data Collection (APDC; dates of admission and discharge, diagnoses codes, procedure codes); and the National Death Index (NDI; month and year of death, set to the last day of the month). Medicare enrollment, PBS, and NDI data were provided by the Australian Institute of Health and Welfare; The Centre for Health Record Linkage conducted the linkage of cancer notifications from the NSWCR and APDC.^13^ The observation period in the NSWCR was January 1982 through December 2019; PBS, APDC, and NDI data were available from January 2012 through March 2022.

### Study design and population

Our population-based, retrospective cohort study included all adults (≥18 years) with a first cancer diagnosis (no other prior cancer diagnoses) of CRC (ICD10 codes C18.x, C19.x, and C20.x) from 2013 through March 2017. We stratified our cohort based on extent of disease spread at diagnosis and studied outcomes within groups of people with (1) localized, (2) regional, and (3) metastatic disease. We examined the period of 1 year before and each year for the 5 years following cancer diagnosis to explore medicine use prior to diagnosis, during active cancer therapy, and during the medium-term, post-treatment survivorship period.

### Outcomes and statistical analyses

#### Cohort and treatment characteristics

We used NSWCR data to summarize topography, extent of disease spread at diagnosis, patient sex, age, and residence at diagnosis. We identified receipt of systemic antineoplastic treatment as dispensing of medicines with Anatomical Therapeutic Chemical (ATC) codes beginning “L01” using PBS dispensing data. Chemotherapy regimens for CRC can be delivered safely, effectively and efficiently in outpatient infusion suites, so nearly all antineoplastic treatments for CRC are captured in PBS dispensing records.^14^ We used APDC data to estimate patients’ comorbidity burden during each year around CRC diagnosis using the Charlson Comorbidity Index (CCI) and identify common surgical procedures used to treat CRC—anterior resection, abdominoperineal resection, anal excision, colectomy, endoscopy, hemicolectomy, and polypectomy (see Table S1 for list of relevant ICD-10-AM/Australian Classification of Health Interventions [ACHI] codes). We used NDI data to summarize deaths and the number of people alive during each year of the study period.

#### Prescription medicine use

We counted the number of unique medicines dispensed during each year around CRC diagnosis, excluding antineoplastic medicines (medicines whose ATC code begins, “L01”), and summarized the median and interquartile range (IQR) for each year within each disease spread at diagnosis group. We summarized the 10 most commonly dispensed medicines in each year around CRC diagnosis using the third level ATC classification (e.g., N02A). For purposes of medicine counts we separated all combination products into their constituent active ingredients.

#### Polypharmacy

We defined polypharmacy as the concomitant exposure to 5 or more medicines during at least one 90-day quarter in each year around date of CRC diagnosis. We summarized the number of people experiencing polypharmacy at any time and within each year (12 months) around diagnosis. To determine concomitant medicine exposure, we estimated duration of medicine exposure using existing methods for Australian dispensing data.^15^^,^^16^ As with raw medicine dispensing counts, we split combination products according to their active ingredients and counted the unique number of medicines each person was exposed to at the midpoint of each quarter around primary CRC diagnosis. We measured all medicine use at the fifth level ATC classification code, chemical substance (e.g., N02AA01).

#### Factors associated with polypharmacy

We used logistic regression to compute adjusted odds ratios (ORs) and 95% confidence intervals (CIs) for the association between patient, disease, and treatment factors and polypharmacy. Within each disease spread at diagnosis group, we examined the factors associated with experiencing polypharmacy at 3 landmark periods: 12 months prior to CRC diagnosis until diagnosis (baseline); between 13- and 24-month following diagnosis (Year 2); and between 49- and 60-month following diagnosis (Year 5).^10^^,^^17^ The first analysis included all patients in our cohort; the latter two analyses included only those patients alive for at least part of the landmark period. For instance, patients included in the Year 5 analysis either survived until the censor date (60 months from diagnosis) or they died between 49 and 60 months from diagnosis.

All models were adjusted for sex, age at diagnosis, cancer site, receipt of surgery, receipt of antineoplastic treatment, CCI score, urbanicity of residence at diagnosis (Major Cities, Inner Regional, Outer Regional/Remote) based on the Australian Bureau of Statistics (ABS) Australian Statistical Geography Standard (ASGS) Remoteness Areas 2016,^18^ quintile of area-based disadvantage of residence at diagnosis based on the ABS Index of Relative Socioeconomic Disadvantage (IRSD) 2016,^19^ and year of diagnosis. We assessed receipt of antineoplastic treatment, surgery, and CCI score using all records prior to each landmark point. For the baseline model, we assessed CCI score from 1 year prior to 30 days following diagnosis. As no patients received antineoplastic therapy prior to CRC diagnosis, we omitted this covariate from the baseline models. All analyses were performed using R v4.1.

## Results

We observed 19 056 people diagnosed with CRC between 2013 and 2017; 6797 (35%), 8482 (45%), and 3777 (20%) were diagnosed with localized, regional, and metastatic disease, respectively (Table 1). Most people, across all levels of disease spread at diagnosis were male (51%-53% within each group), and median age at diagnosis ranged from 70 to 71 years. The majority of people across all disease spread at diagnosis groups were diagnosed with colon cancer.

**Table 1:** Demographic and disease characteristics of the cohort, stratified by extent of disease spread at diagnosis.

	Local disease	Regional disease	Metastatic disease
***N* (%)**	6797 (100)	8482 (100)	3777 (100)
**Female, *n* (%)**	3221 (47)	4094 (48)	1767 (47)
**Median age at diagnosis (IQR)**	70 (60, 78)	71 (61, 79)	70 (59, 79)
**Age groups, *n* (%)**			
** 18-49**	734 (11)	721 (9)	434 (12)
** 50-64**	1593 (23)	2095 (25)	1005 (27)
** 65-74**	2107 (31)	2464 (29)	970 (26)
** 75+**	2363 (35)	3202 (38)	1368 (36)
**Cancer type, *n* (%)**			
** Colon cancer**	4456 (66)	1985 (23)	2631 (70)
** Rectal cancer**	2341 (34)	2270 (27)	1146 (30)
**Geographic area of residence, *n* (%)**			
** Inner regional**	1743 (25)	1812 (23)	842 (22)
** Major city**	4526 (67)	5380 (70)	2615 (69)
** Outer regional/remote**	528 (8)	569 (7)	320 (9)
**Relative socioeconomic advantage, *n* (%)**			
** SEIFA Quintile 1**	1380 (20)	1751 (23)	781 (23)
** SEIFA Quintile 2**	1733 (25)	1887 (24)	863 (26)
** SEIFA Quintile 3**	1327 (20)	1518 (20)	684 (21)
** SEIFA Quintile 4**	954 (14)	1053 (13)	429 (13)
** SEIFA Quintile 5**	1403 (21)	1552 (20)	586 (17)

Few people with localized disease (18%) received systemic antineoplastic medicines, while a majority of those with regional (54%) and metastatic (65%) disease were dispensed antineoplastics (Table 2). Surgeries for CRC were common, with 83% of people with localized and regional disease, and 50% with metastatic disease having a surgical record. The majority of people with localized (87%) and regional (78%) disease spread at diagnosis were alive at the end of the 5-year, postdiagnosis observation period, while most people with metastatic disease (73%) had died within 5 years of the CRC diagnosis.

**Table 2:** Postdiagnosis treatments and survival outcomes, stratified by extent of disease spread at diagnosis.

	Local disease	Regional disease	Metastatic disease
**Dispensed antineoplastics through the PBS, *n* (%)**	1257 (18)	4609 (54)	2453 (65)
**Received surgery, *n* (%)**	5623 (83)	7025 (83)	1875 (50)
** Anterior resection**	658 (10)	113 (15)	155 (4)
** Abdominoperineal resection**	247 (4)	31 (4)	62 (2)
** Anal excision**	133 (2)	^a^	6 (0)
** Colectomy**	91 (1)	22 (3)	50 (1)
** Endoscopy**	194 (3)	23 (3)	35 (1)
** Hemicolectomy**	2476 (36)	310 (42)	1194 (32)
** Polypectomy**	4007 (59)	449 (61)	756 (20)
**Died during follow-up, *n* (%)**	851 (13)	1834 (22)	2739 (73)
** Alive at 1 year following diagnosis**	6406 (94)	7824 (92)	2055 (54)
** Alive at 2 years following diagnosis**	6210 (91)	7343 (87)	1447 (38)
** Alive at 3 years following diagnosis**	6096 (90)	7018 (83)	1185 (31)
** Alive at 4 years following diagnosis**	5997 (88)	6797 (80)	1086 (29)
** Alive at 5 years following diagnosis**	5946 (87)	6648 (78)	1038 (27)

a Cells with counts <6 and those that may allow counts of <6 to be inferred have been suppressed per ethical conditions of the study.

**Figure 1.**
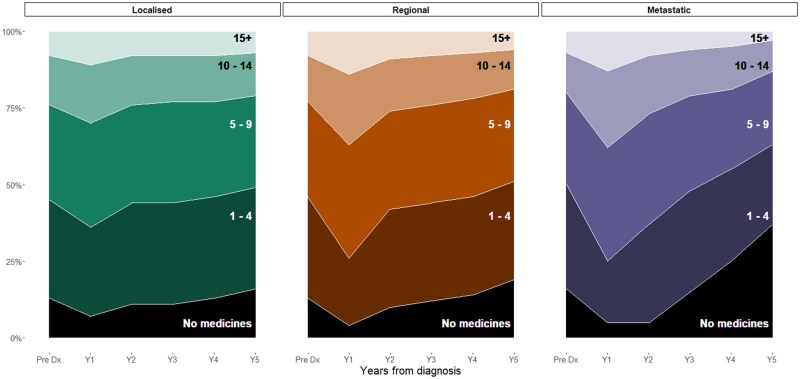
Proportion of each subcohort dispensed *N* number of medicines during each year from CRC diagnosis. Stratified by extent of disease spread at diagnosis.

The median number of unique medicines dispensed in the year prior to CRC diagnosis was 6, regardless of disease spread at diagnosis (Table S2). We observed the largest proportion, and highest number, of medicines dispensed in the 12-month following CRC diagnosis (Year 1) with 30%, 37%, and 38% of people with local, regional, and metastatic disease spread at diagnosis dispensed 10 or more unique medicines respectively during this period (Figure 1 and Table S2). The median number of medicines dispensed also increased to 8 for people with regional and metastatic disease, and to 7 for people with localized spread at diagnosis.

Overall, medicines for the cardiovascular system including lipid modifying agents (i.e., statins), angiotensin II receptor blockers (ARBs), and ACE inhibitors were most commonly dispensed (Figure S1, see online supplementary material for a color version of this figure) to people with localized and regional disease spread at diagnosis across the study period; antiemetics, antinausea medicines, and analgesics comprised the majority of dispensing for people with metastatic disease. Antiemetics, antinausea medicines, and medicines used to treat acid disorders were most commonly dispensed to during the 12-month following diagnosis for all people. Opioid and non-opioid analgesics, as well as antidepressant medicines were most commonly dispensed to all people between 13 and 24 months from diagnosis (Year 2) and remained a large proportion of dispensing for people with regional and metastatic disease spread at diagnosis throughout the remainder of the study period.

**Figure 2.**
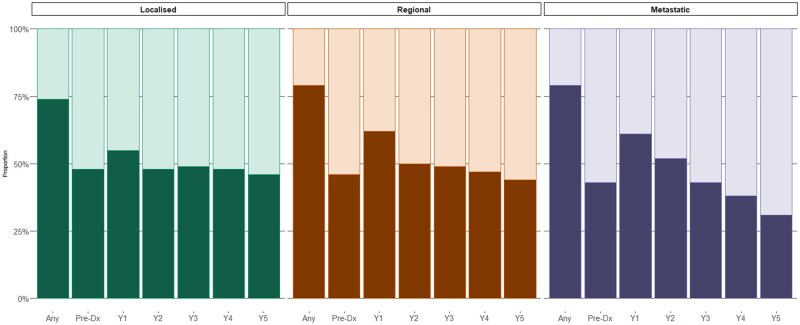
Proportion of each subcohort experiencing polypharmacy (concomitant use of 5 or more medicines; darker shading) at any time during the study period and at any time during each year from CRC diagnosis. Stratified by extent of disease spread at diagnosis.

**Figure 3.**
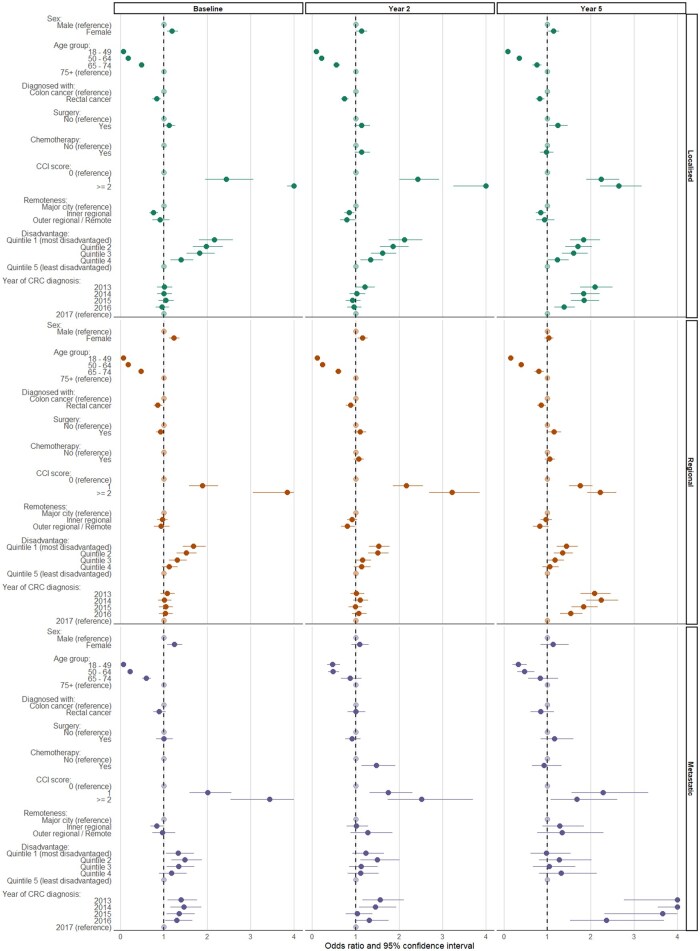
Adjusted odds ratios (points) and 95% confidence intervals (lines) for factors associated with experiencing polypharmacy during the year preceding CRC diagnosis; 2 years following diagnosis; and 5 years following diagnosis. *X*-axis is truncated at OR = 4 for scale. Stratified by extent of disease spread at diagnosis.

Overall, 74%, 79%, and 79% of people with localized, regional, and metastatic disease, respectively, experienced polypharmacy at any time during the study period (Figure 2). Generally, the proportion experiencing polypharmacy during each year around CRC diagnosis ranged between 40% and 50% across all disease spread at diagnosis groups, with a notable increase during the 12-month following CRC diagnosis (55%, 62%, and 61% of people with localized, regional, and metastatic disease, respectively). The factors associated with an increased likelihood of polypharmacy during the 12 months preceding CRC diagnosis included female sex (females more likely experience polypharmacy than males), age group (people < 75 years significantly less likely to experience polypharmacy than those 75 years and older), CCI score (score of ≥1 significantly more likely to experience polypharmacy), and socioeconomic disadvantage (more disadvantaged areas more likely to experience polypharmacy; Figure 3 and Table S3).

In the period 13-24 months from diagnosis, sex, age, CCI score, site (rectal cancer diagnosis significantly less likely to experience polypharmacy), and socioeconomic disadvantage were associated with increased likelihood of polypharmacy for people with localized and regional disease spread at diagnosis (Figure 3 and Table S3). Among people with metastatic disease, age, CCI score, and receipt of antineoplastic treatment (more likely to experience polypharmacy) were associated with increased likelihood of polypharmacy. In the period 49-60 months from diagnosis, age, CCI score, rectal cancer diagnosis, and socioeconomic disadvantage were associated with polypharmacy across nearly all disease spread at diagnosis groups, and year of diagnosis was also significantly associated with polypharmacy at this time (diagnoses before 2017 more likely to experience polypharmacy).

## Discussion

Our study highlights that polypharmacy is common among people diagnosed with CRC, with many taking 10 or more medicines before and after CRC diagnosis. To our knowledge, this is the first study to explore polypharmacy in Australians with CRC. Our findings highlight that medicine use is dynamic, with the number of medicines dispensed increasing for our cohort during the year following diagnosis, likely coinciding with primary cancer therapies, before returning to pre-diagnosis levels for most people.

In general, medicine use in our cohort of people with CRC was greater than that of the broader Australian population. Previous research has reported Australians aged under 50 years use an average of <1 medicine while those aged over 50 use an average ranging from 2 to 5 medicines, depending on age.^15^ The median number of unique medicines dispensed to our cohort was never lower than 6, and 75% of people with localized, regional and metastatic disease were dispensed at least 3 medicines during each year around CRC diagnosis. Similarly, the proportions of people across all levels of disease spread at diagnosis experiencing polypharmacy at any time and within each year around diagnosis were markedly higher than estimates in the entire Australian population (21%)^15^ and above estimates reported in Australians 70 years and older (36%).^20^ This suggests that people with CRC are more likely to have multimorbidity and/or be at higher risk of polypharmacy than the general population prior to their CRC diagnosis, and this polypharmacy amplifies with cancer diagnosis and persists into survivorship.

Our estimates of polypharmacy prevalence in people with CRC—both at any time and yearly—were in-line with those reported from previous studies in all cancers (range: 46%-64%).^21–23^ However, compared with previous studies reporting polypharmacy rates specifically in people with CRC and using the same polypharmacy definition (5 or more medicines), our estimates were generally larger. One large, population-based study examined polypharmacy in people with Stage 0-III disease during the year following diagnosis and reported a rate of 35%, well below our estimates of 55% and 61% in people with localized and regional disease spread at diagnosis, respectively, during the same time period.^6^ Another examined rates between 2 and 3 years from diagnosis and reported 24% experiencing polypharmacy; nearly half that of our estimates of 49%, 43%, and 49% in people with localized, regional, and metastatic spread at diagnosis, respectively, during that time period.^10^ These differences may be explained partially by different study inclusion/exclusion criteria, but each study similarly ascertained medicine use based on similar administrative dispensing claims. Two studies measuring medicine use at CRC diagnosis reported polypharmacy rates both larger (71%)^7^ and in-line with our estimates (55%) from the year to diagnosis.^9^

Polypharmacy has been associated with a reduced quality of life (QoL) amongst older people in general,^24^ those living with chronic disease,^25^ and specifically amongst cancer survivors.^26^ Our study found relatively high survival rates, with 87% and 78% of people with localized and regional disease spread at diagnosis, respectively, alive at the censor date. Almost 50% of these people experienced polypharmacy in years 2 through 5 of the study period (e.g., after active CRC treatment). Such high rates of polypharmacy are a particular cause for concern for patients with localized and regional disease, many of whom will be considered cured of CRC, as it indicates persistent sequelae of cancer treatment that are likely to impact on patients’ quality of life and increase their risk of non-cancer mortality.

The types of medicines driving polypharmacy in the post-treatment survivorship phase illustrates patients’ comorbidities and health concerns. Older people routinely take a number of medications for chronic conditions and, as CRC is predominantly a disease of advanced age, the types of medicines dispensed to our cohort were in-line with those used by the wider Australian population^15^^,^^16^—cardiac medicines, such as statins and antihypertensives, as well as medicines to treat peptic ulcer/GORD (e.g., proton pump inhibitors). The rates of cardiovascular medicine dispensing among people with CRC in our study are comparable to the general Australian population over 50 years of age.^15^ This is surprising given the cardiotoxic potential of some CRC treatments, the overlapping risk factors for developing CRC and cardiovascular disease (such as inactivity, excess body weight and smoking), and the increased rates of cardiovascular disease among people with cancer. It may be the case that increased cardiovascular risk following CRC is not yet identified and/or treated within the 5-year postdiagnosis timeframe of our study. As the use of cardiotoxic cancer medicines and the numbers of Australians exposed to cancer treatments with cardiotoxic potential increases,^27^ early identification and management of cardiovascular risk factors will be crucial in reducing preventable cardiovascular disease and mortality among people with CRC.

The composition of the most dispensed medicines changed slightly during the year following diagnosis, reflecting increased dispensing of supportive medicines, such as antiemetics, painkillers, and antidepressants. Notably, opioids, other analgesics, and antidepressants remained amongst the most commonly dispensed medicines throughout the postactive treatment survivorship period. Psychotropic polypharmacy specifically has also been associated with reduced health-related QoL amongst cancer survivors.^28^ The increased use of antidepressants is consistent with studies demonstrating higher risks of anxiety and depression in long-term cancer survivors compared with non-cancer controls.^29^

Perhaps unsurprisingly, we found that age and higher comorbidity burden were consistently associated with an increased likelihood of experiencing polypharmacy at all landmark points. Our finding for higher comorbidity burden aligns with those from previous studies.^21^^,^^22^ Interestingly, while the prevalence of polypharmacy is known to increase with age,^15^^,^^21^ age was not significantly associated with polypharmacy in these studies that included both patients of all ages^21^ and those 70 years and older.^22^ This suggests that medicine use itself may be a reasonable proxy for health status/comorbidity burden. Age is also an important consideration in CRC because, while there is less evidence that polypharmacy is problematic in younger patients, incidence of early-onset CRC has grown dramatically over the past 4 decades.^4^ People with early-onset CRC comprised a small proportion of our overall cohort (10%), but more research is warranted into the patterns of care during survivorship for this growing patient population.

### Strengths and limitations

Our study used a large, population-based data collection to describe multiple medicine use, the prevalence of polypharmacy, and the factors associated with polypharmacy in a large cohort of people diagnosed with CRC. Our data do not include clinical measures, and measures such as comorbidity burden were ascertained via hospitalization records. These estimates likely underestimate the true extent of clinical phenomena. Our dispensing data likely capture the overwhelming majority of prescription medicines dispensed to our cohort during the study period. However, they do not contain information on the intended duration of treatment, and we estimated treatment duration based on dispensing records. Our estimates of polypharmacy may be slightly higher than the true rates as we do not observe exactly how people are using their dispensed medicines and cannot directly ascertain when they might discontinue medications. These data also do not contain information on medicines dispensed in-hospital or over-the-counter, medicines received through clinical trials or special access programs, or the multitude of complementary and alternative medicines taken by cancer patients, and, in this way, our estimates may slightly underestimate polypharmacy prevalence.

## Conclusions

Our findings suggest that polypharmacy is highly prevalent among Australians with CRC and potentially higher than in similar international jurisdictions. While medicine use remained fairly stable following active cancer treatment, the number of medicines dispensed to our cohort was higher than those used in the general Australian population. For patients treated with curative intent, higher-than-expected rates of polypharmacy are particularly concerning, as they indicate excess morbidity and impaired quality of life among survivors who have been cured of cancer but continue to experience poorer health than the general population.

## Supplementary Material

oyaf380_Supplementary_Data

## Data Availability

Direct access to the data and analytical files to other individuals or authorities is not permitted without the express permission of the approving human research ethics committees and data custodians.
