# Supplementary material for: Polypharmacy among people diagnosed with colorectal cancer in Australia: a population-based cohort study
Source: Oncologist. 2025 Dec 9;31(5):oyaf380. doi: 10.1093/oncolo/oyaf380 (PMC13138378; doi:10.1093/oncolo/oyaf380)
Supplement: oyaf380_Supplementary_Data [file oyaf380_supplementary_data.zip › Revised Supplementary Figures.docx]

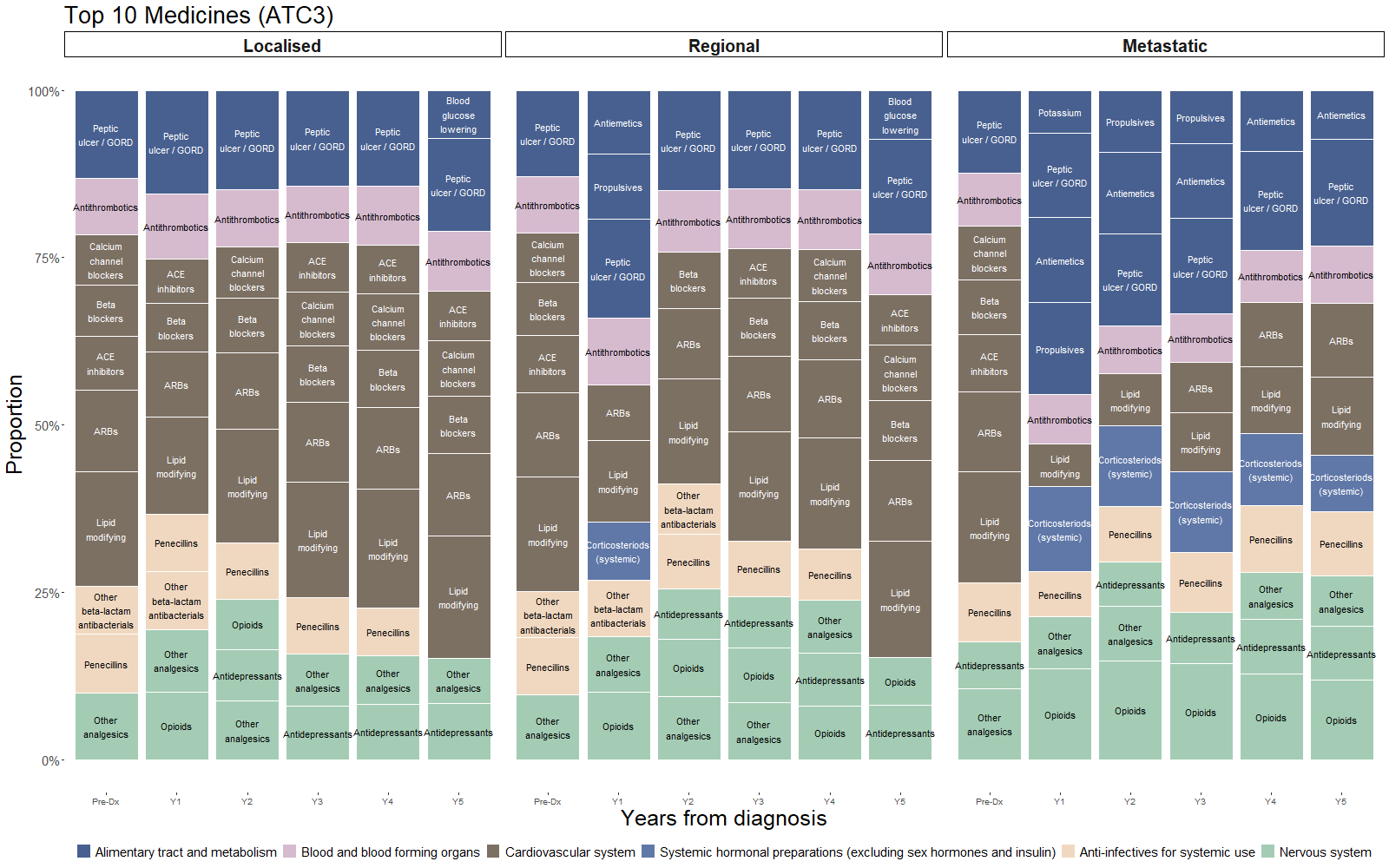


Supplementary Figure S1. Top 10 most dispensed medicine groups (ATC level 3, pharmacological subgroups) during each year from CRC diagnosis. The bars show the proportion of dispensings within the top 10 accounted for by each medicine group and colours indicate the anatomical group (ATC level 1) to which each medicine group belongs. Stratified by extent of disease spread at diagnosis.
