# Supplementary material for: Polypharmacy among people diagnosed with colorectal cancer in Australia: a population-based cohort study
Source: Oncologist. 2025 Dec 9;31(5):oyaf380. doi: 10.1093/oncolo/oyaf380 (PMC13138378; doi:10.1093/oncolo/oyaf380)
Supplement: oyaf380_Supplementary_Data [file oyaf380_supplementary_data.zip › Revised Supplementary Tables.docx]

Supplementary Table S1. ICD-10-AM / Australian Classification of Health Interventions (ACHI) codes for surgical procedures of interest.

| **Procedure** | **Codes** |
| --- | --- |
| Anterior resection | 933, 935, 32024-00, 32025-00, 32026-00, 32028-00, 32028-01, 92208-00 |
| Abdominoperineal resection | 32039-00 |
| Anal excision | 32099-00, 32103-00, 32105-00, 32108-00 |
| Colectomy | 913, 934, 936, 32009-00, 32009-01, 32012-00, 32012-01, 32015-00, 32051-00, 32051-01, 32051-02, 32051-03 |
| Endoscopy | 1006, 1007, 1008, 30478-00, 30478-03, 30478-04, 30478-14, 30478-17, 30478-18, 30478-20, 30478-21 |
| Hemicolectomy | 32000-00, 32000-01, 32000-02, 32000-03, 32003-00, 32003-01, 32003-02, 32003-03, 32004-00, 32004-01, 32004-02, 32004-03, 32005-00, 32005-01, 32005-02, 32005-03, 32006-00, 32006-01, 32006-02,  32006-03 |
| Polypectomy | 32078-00, 32081-00, 32093-00, 32087-00 |
|  |  |

Supplementary Table S2. Median and interquartile range (IQR) number of medicines dispensed and the number (%) of people dispensed N medicines during each year from CRC diagnosis. Stratified by extent of disease spread at diagnosis.

|  |  | Year prior to diagnosis | Year 1 | Year 2 | Year 3 | Year 4 | Year 5 |
| --- | --- | --- | --- | --- | --- | --- | --- |
| **Local disease n (%)** | |  |  |  |  |  |  |
| Dispensed medicines,   median (IQR) | | 6 (3, 10) | 7 (4, 11) | 6 (3, 10) | 6 (3, 10) | 6 (3, 10) | 6 (3, 9) |
|  | No medicines | 852 (13) | 458 (7) | 702 (11) | 719 (11) | 818 (13) | 949 (16) |
|  | 1 – 4 medicines | 2,244 (33) | 1,970 (29) | 2,132 (33) | 2,058 (33) | 1,994 (33) | 1,964 (33) |
|  | 5 – 9 medicines | 2,079 (31) | 2,291 (34) | 2,018 (32) | 2,019 (33) | 1,898 (31) | 1,828 (30) |
|  | 10 – 14 medicines | 1,060 (16) | 1,301 (19) | 1,027 (16) | 907 (15) | 927 (15) | 848 (14) |
|  | 15+ medicines | 562 (8) | 777 (11) | 527 (8) | 507 (8) | 459 (8) | 408 (7) |
|  |  |  |  |  |  |  |  |
| **Regional disease** | |  |  |  |  |  |  |
| Dispensed medicines,   median (IQR) | | 6 (3, 10) | 8 (5, 12) | 6 (3, 10) | 6 (3, 10) | 6 (3, 10) | 6 (3, 9) |
|  | No medicines | 1,105 (13) | 297 (4) | 769 (10) | 860 (12) | 1,021 (14) | 1,258 (19) |
|  | 1 – 4 medicines | 2,817 (33) | 1,838 (22) | 2,523 (32) | 2,370 (32) | 2,226 (32) | 2,159 (32) |
|  | 5 – 9 medicines | 2,647 (31) | 3,205 (38) | 2,528 (32) | 2,390 (32) | 2,212 (32) | 2,073 (30) |
|  | 10 – 14 medicines | 1,271 (15) | 1,975 (23) | 1,345 (17) | 1,139 (16) | 1,048 (15) | 884 (13) |
|  | 15+ medicines | 642 (8) | 1,167 (14) | 659 (8) | 584 (8) | 511 (7) | 423 (6) |
|  |  |  |  |  |  |  |  |
| **Metastatic disease** | |  |  |  |  |  |  |
| Dispensed medicines,   median (IQR) | | 6 (3, 9) | 8 (5, 12) | 7 (3, 10) | 6 (3, 9) | 6 (3, 10) | 6 (3, 9) |
|  | No medicines | 656 (17) | 200 (5) | 97 (5) | 213 (15) | 303 (25) | 397 (37) |
|  | 1 – 4 medicines | 1,272 (34) | 766 (20) | 654 (32) | 471 (33) | 355 (30) | 281 (26) |
|  | 5 – 9 medicines | 1,115 (30) | 1,351 (36) | 747 (36) | 456 (31) | 304 (26) | 260 (24) |
|  | 10 – 14 medicines | 478 (13) | 954 (25) | 381 (19) | 216 (15) | 164 (14) | 113 (10) |
|  | 15+ medicines | 256 (7) | 506 (13) | 176 (8) | 91 (6) | 59 (5) | 35 (3) |
|  |  |  |  |  |  |  |  |

Supplementary Table S3. Adjusted odds ratios and 95% confidence intervals for factors associated with experiencing polypharmacy during the year preceding CRC diagnosis; two years following diagnosis; and five years following diagnosis.

|  | **Local disease** | | | **Regional disease** | | | **Metastatic disease** | | |  |  |
| --- | --- | --- | --- | --- | --- | --- | --- | --- | --- | --- | --- |
| **Local disease** | **Baseline** | **Year 2** | **Year 5** | **Baseline** | **Year 2** | **Year 5** | **Baseline** | **Year 2** | **Year 5** |  |  |
| Sex: |  |  |  |  |  |  |  |  |  |  |  |
| Male (reference) | 1.00 (-) | 1.00 (-) | 1.00 (-) | 1.00 (-) | 1.00 (-) | 1.00 (-) | 1.00 (-) | 1.00 (-) | 1.00 (-) |  |  |
| Female | 1.19 (1.07 – 1.33) | 1.14  (1.02 – 1.27) | 1.14  (1.02 – 1.28) | 1.23 (1.12 – 1.36) | 1.16 (1.05 – 1.28) | 1.03 (0.92 – 1.14) | 1.24 (1.07 – 1.43) | 1.09 (0.91 – 1.30) | 1.13 (0.85 – 1.50) |  |  |
|  |  |  |  |  |  |  |  |  |  |  |  |
| Age group: |  |  |  |  |  |  |  |  |  |  |  |
| 18 - 49 | 0.06 (0.04 – 0.07) | 0.09 (0.07 – 0.11) | 0.09 (0.07 – 0.12) | 0.06 (0.04 – 0.08) | 0.12 (0.10 – 0.15) | 0.16 (0.13 – 0.21) | 0.06 (0.04 – 0.09) | 0.47 (0.35 – 0.64) | 0.33 (0.20 – 0.52) |  |  |
| 50 – 64 | 0.17 (0.15 – 0.20) | 0.22 (0.19 – 0.25) | 0.35 (0.30 – 0.41) | 0.17 (0.15 – 0.20) | 0.24 (0.21 – 0.27) | 0.40 (0.35 – 0.46) | 0.22 (0.18 – 0.26) | 0.48 (0.37 – 0.62) | 0.47 (0.31 – 0.70) |  |  |
| 65 – 74 | 0.48 (0.43 – 0.55) | 0.56 (0.49 – 0.64) | 0.76 (0.66 – 0.87) | 0.47 (0.42 – 0.52) | 0.60 (0.53 – 0.68) | 0.80 (0.71 – 0.92) | 0.59 (0.50 – 0.70) | 0.87 (0.67 – 1.14) | 0.84 (0.56 – 1.25) |  |  |
| 75+ | 1.00 (-) | 1.00 (-) | 1.00 (-) | 1.00 (-) | 1.00 (-) | 1.00 (-) | 1.00 (-) | 1.00 (-) | 1.00 (-) | - | |
|  |  |  |  |  |  |  |  |  |  |  |  |
| Diagnosed with: |  |  |  |  |  |  |  |  |  |  |  |
| Colon cancer   (reference) | 1.00 (-) | 1.00 (-) | 1.00 (-) | 1.00 (-) | 1.00 (-) | 1.00 (-) | 1.00 (-) | 1.00 (-) | 1.00 (-) | |  |
| Rectal cancer | 0.83 (0.74 – 0.93) | 0.74 (0.66 – 0.84) | 0.83 (0.74 – 0.94) | 0.86 (0.77 – 0.96) | 0.88 (0.78– 0.98) | 0.86 (0.77 – 0.97) | 0.89 (0.76 – 1.04) | 1.00 (0.82 – 1.22) | 0.85 (0.62 – 1.16) |  |  |
|  |  |  |  |  |  |  |  |  |  |  |  |
| Surgery: |  |  |  |  |  |  |  |  |  |  |  |
| No (reference) | 1.00 (-) | 1.00 (-) | 1.00 (-) | 1.00 (-) | 1.00 (-) | 1.00 (-) | 1.00 (-) | 1.00 (-) | 1.00 (-) | |  |
| Yes | 1.12 (1.00 – 1.26) | 1.14 (0.97 – 1.33) | 1.24 (1.04 – 1.47) | 0.92 (0.82– 1.02) | 1.10 (0.95– 1.25) | 1.15 (0.99 – 1.32) | 1.00 (0.82 – 1.21) | 0.92 (0.76 – 1.11) | 1.17 (0.85 – 1.60) |  |  |
|  |  |  |  |  |  |  |  |  |  |  |  |
| Antineoplastic treatment |  |  |  |  |  |  |  |  |  |  |  |
| No (reference) | 1.00 (-) | 1.00 (-) | 1.00 (-) | 1.00 (-) | 1.00 (-) | 1.00 (-) | 1.00 (-) | 1.00 (-) | 1.00 (-) | |  |
| Yes | ^a^ | 1.14 (0.98 – 1.34) | 0.98 (0.84 – 1.14) | ^a^ | 1.07 (0.96 – 1.19) | 1.06 (0.95 – 1.18) | ^a^ | 1.48 (1.14 – 1.92) | 0.92 (0.65 – 1.33) |  |  |
|  |  |  |  |  |  |  |  |  |  |  |  |
| CCI score: |  |  |  |  |  |  |  |  |  |  |  |
| 0 (reference) | 1.00 (-) | 1.00 (-) | 1.00 (-) | 1.00 (-) | 1.00 (-) | 1.00 (-) | 1.00 (-) | 1.00 (-) | 1.00 (-) | |  |
| 1 | 2.44 (1.95 – 3.06) | 2.43 (2.02 – 2.93) | 2.24 (1.90 – 2.65) | 1.89 (1.58 – 2.25) | 2.17 (1.86 – 2.55) | 1.76 (1.51 – 2.04) | 2.01 (1.59 – 2.56) | 1.75 (1.32 – 2.31) | 2.28 (1.56 – 3.33) |  |  |
| >2 | 5.17 (3.84 – 7.09) | 4.08 (3.26 – 5.15) | 2.65 (2.22 – 3.17) | 3.84 (3.05 – 4.86) | 3.22 (1.70 – 3.86) | 2.32 (1.92 – 2.59) | 3.44 (2.53 – 4.74) | 2.52 (1.74 – 3.70) | 1.68 (1.08 – 2.62) |  |  |
|  |  |  |  |  |  |  |  |  |  |  |  |
| Remoteness: |  |  |  |  |  |  |  |  |  |  |  |
| Major city   (reference) | 1.00 (-) | 1.00 (-) | 1.00 (-) | 1.00 (-) | 1.00 (-) | 1.00 (-) | 1.00 (-) | 1.00 (-) | 1.00 (-) | |  |
| Inner regional | 0.76 (0.67 – 0.87) | 0.85 (0.74 – 0.97) | 0.85 (0.74 – 0.97) | 0.96 (0.85 – 1.09) | 0.92 (0.81 – 1.04) | 0.97 (0.85 – 1.11) | 0.83 (0.69 – 1.00) | 1.02 (0.80 – 1.29) | 1.29 (0.89 – 1.85) |  |  |
| Outer regional /   Remote | 0.91 (0.74 – 1.13) | 0.80 (0.64 – 0.99) | 0.94 (0.75 – 1.17) | 0.93 (0.77 – 1.13) | 0.81 (0.66 – 0.98) | 0.83 (0.67 – 1.03) | 0.96 (0.73 – 1.26) | 1.28 (0.89 – 1.85) | 1.34 (0.77 – 2.30) |  |  |
|  |  |  |  |  |  |  |  |  |  |  |  |
| Disadvantage: |  |  |  |  |  |  |  |  |  |  |  |
| Quintile 1 (most   disadvantaged) | 2.16 (1.81 – 2.59) | 2.12 (1.76 – 2.54) | 1.84 (1.53 – 2.22) | 1.68 (1.44 – 1.96) | 1.53 (1.31 – 1.78) | 1.44 (1.22 – 1.70) | 1.33 (1.05 – 1.69) | 1.24 (0.93 – 1.65) | 0.98 (0.62 – 1.54) |  |  |
| Quintile 2 | 1.98 (1.67 – 2.36) | 1.86 (1.56 – 2.22) | 1.70 (1.42 – 2.03) | 1.51 (1.29 – 1.76) | 1.51 (1.29 – 1.76) | 1.35 (1.15 – 1.59) | 1.48 (1.17 – 1.88) | 1.50 (1.12 – 2.02) | 1.28 (0.81 – 2.02) |  |  |
| Quintile 3 | 1.82 (1.53 – 2.17) | 1.62 (1.36 – 1.94) | 1.61 (1.34 – 1.93) | 1.31 (1.12 – 1.53) | 1.16 (0.99 – 1.36) | 1.18 (1.00 – 1.39) | 1.34 (1.06 – 1.70) | 1.13 (0.85 – 1.51) | 1.05 (0.67 – 1.65) |  |  |
| Quintile 4 | 1.39 (1.15 – 1.68) | 1.35 (1.11 – 1.63) | 1.23 (1.01 – 1.50) | 1.12 (0.95 – 1.32) | 1.14 (0.96 – 1.35) | 1.06 (0.89 – 1.27) | 1.17 (0.89 – 1.52) | 1.12 (0.82 – 1.53) | 1.32 (0.82 – 2.14) |  |  |
| Quintile 5 (least   disadvantaged) | 1.00 (-) | 1.00 (-) | 1.00 (-) | 1.00 (-) | 1.00 (-) | 1.00 (-) | 1.00 (-) | 1.00 (-) | 1.00 (-) |  |  |
|  |  |  |  |  |  |  |  |  |  |  |  |
| Year of CRC diagnosis: |  |  |  |  |  |  |  |  |  |  |  |
| 2013 | 1.01 (0.85 – 1.20) | 1.21 (1.02 – 1.44) | 2.10 (1.76 – 2.51) | 1.07 (0.92 – 1.25) | 1.02 (0.88 – 1.20) | 2.09 (1.77 – 2.46) | 1.39 (1.09 – 1.77) | 1.56 (1.16 – 2.11) | 4.42 (2.77 – 7.14) |  |  |
| 2014 | 1.00 (0.84 – 1.19) | 1.03 (0.86 – 1.23) | 1.84 (1.54 – 2.21) | 1.01 (0.87 – 1.17) | 1.10 (0.95 – 1.29) | 2.24 (1.90 – 2.64) | 1.46 (1.15 – 1.86) | 1.45 (1.08 – 1.94) | 5.59 (3.55 – 8.94) |  |  |
| 2015 | 1.04 (0.88 – 1.23) | 0.93 (0.78 – 1.11) | 1.85 (1.55 – 2.20) | 1.04 (0.89 – 1.21) | 0.99 (0.84 – 1.15) | 1.83 (1.56 – 2.16) | 1.35 (1.06 – 1.71) | 1.04 (0.77 – 1.39) | 3.66 (2.32 – 5.83) |  |  |
| 2016 | 0.95 (0.81 – 1.12) | 0.96 (0.81 – 1.14) | 1.38 (1.17 – 1.64) | 1.03 (0.89 – 1.21) | 1.07 (0.92 – 1.26) | 1.54 (1.30 – 1.81) | 1.30 (1.03 – 1.66) | 1.31 (0.98 – 1.76) | 2.36 (1.53 – 3.69) |  |  |
| 2017 (reference) | 1.00 (-) | 1.00 (-) | 1.00 (-) | 1.00 (-) | 1.00 (-) | 1.00 (-) | 1.00 (-) | 1.00 (-) | 1.00 (-) |  |  |
|  |  |  |  |  |  |  |  |  |  |  |  |

^a^No one had received chemotherapy at baseline and these covariates were excluded from the baseline models.
